# Effect of Intravenous Alteplase on Functional Outcome and Secondary Injury Volumes in Stroke Patients with Complete Endovascular Recanalization

**DOI:** 10.3390/jcm11061565

**Published:** 2022-03-12

**Authors:** Gabriel Broocks, Lukas Meyer, Celine Ruppert, Wolfgang Haupt, Tobias D. Faizy, Noel Van Horn, Matthias Bechstein, Helge Kniep, Sarah Elsayed, Andre Kemmling, Ewgenia Barow, Jens Fiehler, Uta Hanning

**Affiliations:** 1 Department of Neuroradiology, University Medical Center Hamburg-Eppendorf, 20251 Hamburg, Germany; lu.meyer@uke.de (L.M.); celine@ruppertinheidelberg.de (C.R.); wolfgang.haupt0@gmail.com (W.H.); t.faizy@uke.de (T.D.F.); no.vanhorn@uke.de (N.V.H.); m.bechstein@uke.de (M.B.); h.kniep@uke.de (H.K.); s.elsayed@uke.de (S.E.); fiehler@uke.de (J.F.); u.hanning@uke.de (U.H.); 2 Department of Neuroradiology, Philipps-University Marburg, 35037 Marburg, Germany; akemmling@gmail.com; 3 Department of Neuroradiology, University Medical Center Schleswig-Holstein, Campus Lübeck, 23538 Lübeck, Germany; 4 Department of Neurology, University Medical Center Hamburg-Eppendorf, 20251 Hamburg, Germany; e.barow@uke.de

**Keywords:** stroke, thrombectomy, thrombolysis

## Abstract

Intravenous thrombolytic therapy with alteplase (IVT) is a standard of care in ischemic stroke, while recent trials investigating direct endovascular thrombectomy (EVT) approaches showed conflicting results. Yet, the effect of IVT on secondary injury volumes in patients with complete recanalization has not been analyzed. We hypothesized that IVT is associated with worse functional outcome and aggravated secondary injury volumes when administered to patients who subsequently attained complete reperfusion after EVT. Anterior circulation ischemic stroke patients with complete reperfusion after thrombectomy defined as thrombolysis in cerebral infarctions (TICI) scale 3 after thrombectomy admitted between January 2013–January 2021 were analyzed. Primary endpoints were the proportion of patients with functional independence defined as modified Rankin Scale (mRS) score 0–2 at day 90, and secondary injury volumes: Edema volume in follow-up imaging measured using quantitative net water uptake (NWU), and the rate of symptomatic intracerebral hemorrhage (sICH). A total of 219 patients were included and 128 (58%) patients received bridging IVT before thrombectomy. The proportion of patients with functional independence was 28% for patients with bridging IVT, and 34% for patients with direct thrombectomy (*p* = 0.35). The rate of sICH was significantly higher after bridging IVT (20% versus 7.7%, *p* = 0.01). Multivariable logistic and linear regression analysis confirmed the independent association of bridging IVT with sICH (aOR: 2.78, 95% CI: 1.02–7.56, *p* = 0.046), and edema volume (aOR: 8.70, 95% CI: 2.57–14.85, *p* = 0.006). Bridging IVT was associated with increased edema volume and risk for sICH as secondary injury volumes. The results of this study encourage direct EVT approaches, particularly in patients with higher likelihood of successful EVT.

## 1. Introduction

The administration of intravenous recombinant tissue plasminogen activator (IVT) within the first hours after stroke onset is a standard of care in ischemic stroke, and is often used in combination with endovascular thrombectomy (EVT), which has proven to be beneficial to patients with ischemic stroke caused by a large vessel occlusion (LVO) in the anterior circulation [1,2,3,4]. The primary treatment target of IVT and EVT in LVO stroke is vessel recanalization, which has shown to be the main determinant of functional outcome [5,6,7]. Vessel recanalization after IVT alone, however, occurs rarely, depending on vessel size [5]. On the other side, reported recanalization rates in EVT trials increased continuously: 40% in IMS-III, 2013; 60% in MR CLEAN, 2015; 77% in DEFUSE-3, 2018; 86% in ESCAPE-NA1, 2020, respectively [8,9,10], mainly due to better devices and rising experience. IVT has been administered in the majority of the included patients, except DEFUSE-3 with only 10% of patients receiving IVT. Recently, it has been observed that direct EVT was non-inferior compared to patients undergoing EVT with prior IVT with regards to functional outcomes, however, other trials did not observe non-inferiority of direct EVT [11,12]. Consequently, it is discussed controversially to ascertain in which patients to apply IVT, and in particular to investigate factors associated with additional harm [13,14,15]. The increasing rates of vessel recanalization after EVT alone emphasize the need to investigate the effects of IVT on lesion pathophysiology and clinical outcome in patients with complete reperfusion. The purpose of this study was to compare clinical outcomes and secondary injury volumes in patients with versus without IVT and complete EVT. We hypothesized that IVT is associated with worse functional outcome at day 90 and increased secondary injury volumes at follow-up imaging (i.e., symptomatic intracranial hemorrhage and ischemic edema formation) when administered in patients with ischemic stroke who subsequently underwent complete recanalization by EVT with a thromboloysis in cerebral infarction (TICI) of 3.

## 2. Materials and Methods

### 2.1. Patients

All ischemic stroke patients with LVO in the anterior circulation admitted between January 2013–January 2021 at a high-volume tertiary stroke center were consecutively analyzed. Only anonymized data were analyzed after ethical review board approval, and informed consent was waived after review.

The a priori defined inclusion criteria for this study were: (1) ischemic anterior circulation LVO stroke with multimodal CT imaging on admission (non-enhanced CT (NECT), CT angiography (CTA) and CT perfusion (CTP)); (2) occlusion of the intracranial internal carotid artery or proximal middle cerebral artery (M1 segment); (3) known onset of symptoms; (4) complete reperfusion after EVT defined as a Thrombolysis in Cerebral Infarction (TICI) score of 3; (5) absence of intracranial hemorrhage; (6) absence of significant imaging artifacts. TICI rating was determined by the operating neurointerventionalist and validated by a further attending neuroradiologist. Good functional outcomes were defined as modified Rankin Scale (mRS) scores 0–2. The mRS scores were evaluated at the 90-day follow-up by a physician or a trained and certified mRS nurse. sICH was defined according to the second European–Australasian Acute Stroke Study (ECASS II) as presence of intracerebral hemorrhage and a 4-point neurological deterioration on the National Institute of Health Stroke Scale (NIHSS) [16].

### 2.2. Revascularization Protocol

IVT was administered to patients according to current guidelines [17]. Laboratory and conventional clinical inclusion and exclusion criteria for IVT applied. EVT was performed via a femoral artery approach under general anesthesia or conscious sedation. Endovascular procedures were performed by board-certified interventional neuroradiologists. The choice of thrombectomy device was left to the operator.

### 2.3. Image Analysis

The readers were blinded for all clinical data. Image analyses including volumetric and densitometric analysis were performed using commercially available software (Analyze 11.0, Biomedical Imaging Resource, Mayo Clinic, Rochester, MN, USA) by a board-certified experienced neuroradiologist using standardized procedures. Ischemic lesion net water uptake (NWU) was measured as an imaging biomarker to quantify cerebral ischemic edema before treatment on admission CT and on follow-up CT (FCT) 24 h after treatment with a standardized procedure, as previously described in detail [7,18,19,20]. NWU describes ischemic lesion water uptake per volume of brain infarct (i.e., relative proportion of edema) [7,20]. ΔNWU was defined as the additional NWU from admission to FCT for each patient (ΔNWU = NWU*_fct_* − NWU*_admission_*) [7]. Total infarct volume has been defined as the volume of the whole visually evident lesion in FCT and was measured using semiautomatic volumetric segmentation. Edema volume (EV) as a measure of the absolute volume of edema was then determined as described before [21] as the product of the total infarct volume and NWU*_fct_* according to Equation (1) according to Nawabi et al. [21].

Equation (1) [19,21]:

Edema volume *=* Total infarct volume * % − NWU (1)

### 2.4. Statistical Analysis

Univariable distribution of metric variables was described by median and interquartile range (IQR) or means and standard deviation (SD). Categorial variables were compared using *χ*^2^-tests. Shapiro–Wilk tests were used to test for normal distribution. Patients who received bridging IVT were compared to patients who did not receive IVT and directly underwent EVT. Patient characteristics are shown in Table 1. As endpoints, we defined the proportion of patients with functional independence (mRS 0–2), the degree of edema formation (ΔNWU), the occurrence of sICH, and edema volume. To determine the treatment effect of IVT on the aforementioned endpoints, we used inverse-probability weighted regression adjustments using logit outcome and treatment models adjusted for baseline variables (age, NIHSS, ASPECTS, occlusion location, and time from onset to imaging). Secondly, we investigated the association of the independent variables on functional independence and sICH using uni- and multivariable logistic regression analysis, as well as ΔNWU and edema volume using linear regression analysis, respectively. Correlation between all independent variables was tested to exclude multicollinearity.

A statistically significant difference was accepted at a *p*-value of less than 0.05. Analyses were performed using Stata 17.0 (StataMP, StataCorp, College Station, TX, USA).

## 3. Results

### 3.1. Study Cohort

A total of 219 patients were included. The patient characteristics are displayed in Table 1. The median age was 74 (IQR: 63–81) and 106 patients were female (48%). The median NIHSS was 16 (IQR: 11–20) and the median ASPECTS was 7 (IQR: 6–8). The median time from symptom onset to imaging was 3 h (IQR: 1.3–4.0). A total of 128 patients received IVT (58%). The median time from imaging to recanalization was 1.7 h (IQR: 1.4–2.4). After 24 h, the median NIHSS was 11 (IQR: 4–20), and the median mRS at day 90 was 3 (IQR: 2–5).

**Figure 1:**
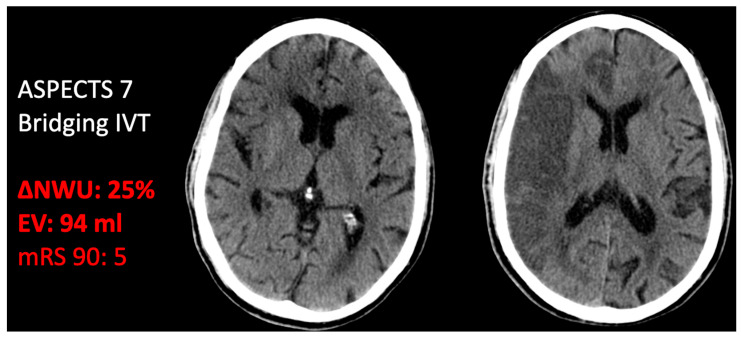
Illustration of a patient receiving bridging intravenous alteplase prior to complete endovascular recanalization. The patient showed significant edema progression with aggravated absolute edema volume in follow-up imaging. ΔNWU reflects the change of the relative edema proportion from admission to follow-up (ΔNWU = % NWU admission − % NWU follow-up) as previously defined [7].

Comparing patients who received IVT to patients who did not receive IVT, there were no significant differences in sex, admission NIHSS (median 16, *p* = 0.66), or ASPECTS (median 6 versus 7, *p* = 0.63). Tendentially, patients with IVT were younger (73 versus 76 years, *p* = 0.06). There were no differences regarding the time from symptom onset to imaging (3.1 versus 2.0 h, *p* = 0.38) and time from imaging to complete reperfusion (1.7 versus 1.8 h, *p* = 0.05) (Table 1). Figure 1 illustrates an example of a patient receiving IVT who develops significant edema formation. In total, 94 patients (43%) showed futile recanalization defined as mRS 4–6 at day 90 despite complete recanalization. Considering early neurological change, 92% of all patients showed a lower NIHSS score at discharge compared to the NIHSS on admission.

### 3.2. Impact of IVT on Clinical Endpoints

**Figure 2:**
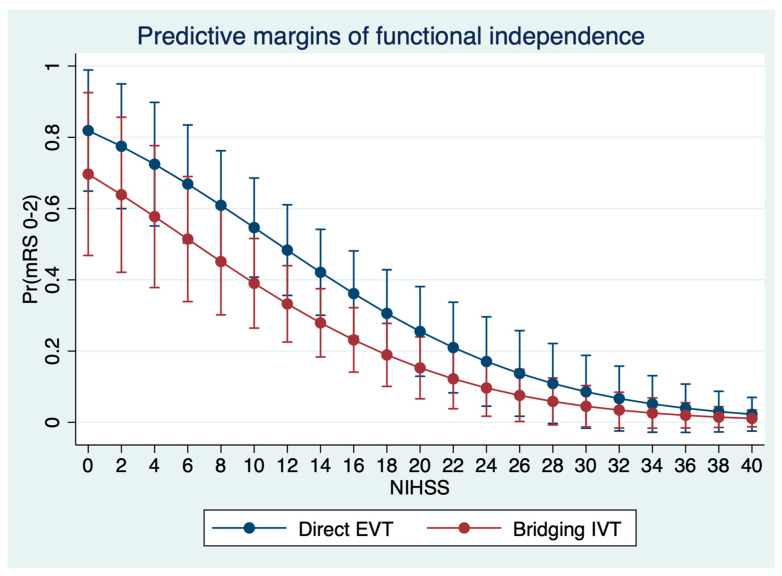
Prediction of functional independence (*y* axis) based on multivariable logistic regression analysis to show the impact of IVT application according to the baseline NIHSS adjusted for age and ASPECTS (Table 2).

Comparing patients with bridging IVT to patients with direct EVT, no differences were observed in the median NIHSS at 24 h (10 versus 13, *p* = 0.75). The median mRS at day 90 was similar: 4 (IQR: 2–5) in patients with bridging IVT, and 3 (IQR: 1–5) in patients with direct EVT (*p* = 0.61). The proportion of patients with functional independence was 28% for patients with bridging IVT and 34% for patients with direct EVT (*p* = 0.35) (Table 1). After regression adjustment, no significant treatment effect of IVT on functional independence as an endpoint was observed, adjusted for age, NIHSS, ASPECTS, occlusion location, and time from onset to imaging (*p* = 0.11), with an adjusted proportion of functional independence of 38% (95% CI: 28–48%) for patients with direct EVT, compared to 28% (95% CI: 19–37%) for patients with bridging IVT. In multivariable logistic regression analysis, IVT was not associated with functional independence, despite a small trend towards reduced probability for good outcome (OR: 0.48, *p* = 0.09). Independent significant predictors were age (OR: 0.93, *p* < 0.001), and NIHSS (OR: 0.85, *p* < 0.001) (Table 2, Figure 2).

### 3.3. Impact of IVT on Secondary Injury Volumes

**Figure 3:**
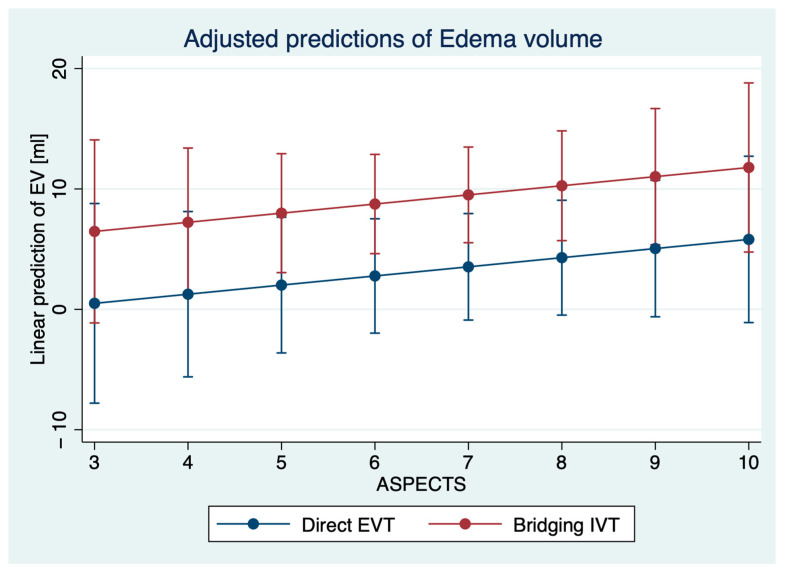
Prediction of edema volume (*y* axis) based on multivariable linear regression analysis to show the impact of IVT application according to the baseline ASPECTS. No further variable was independently associated with EV.

**Figure 4:**
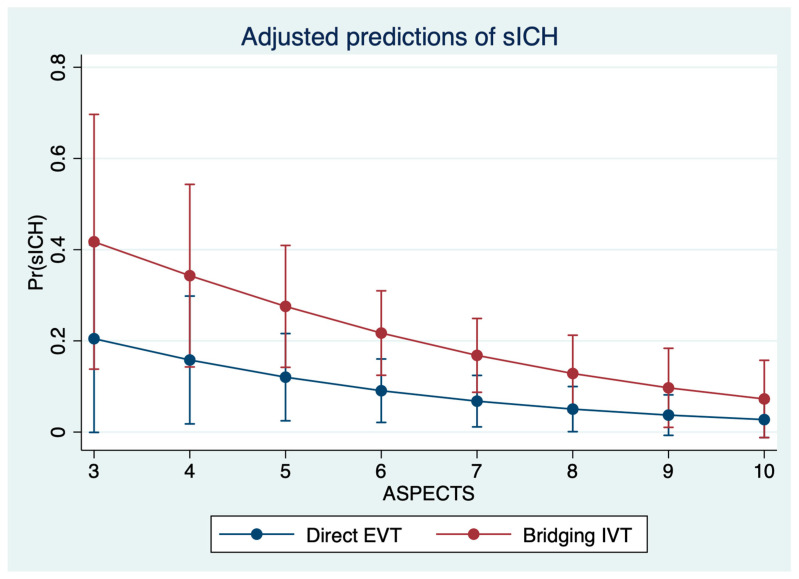
Prediction of symptomatic intracranial hemorrhage (*y* axis) based on multivariable logistic regression analysis to show the impact of IVT application according to the baseline ASPECTS, adjusted for time from onset to imaging.

Patients with bridging IVT had higher total infarct volumes (48 mL versus 37 mL, *p* = 0.04), and higher EV (6 mL versus 4 mL, *p* = 0.039). The rate of sICH was significantly higher in patients with bridging IVT (26% versus 7%, *p* = 0.01). After regression adjustment, IVT had a significant effect on the occurrence of sICH (*p* = 0.029) with an adjusted proportion of sICH of 7.8% (95% CI: 1.7–13.8%) in direct EVT patients versus 19.0% (95% CI: 10.9–27.1%), adjusted for age, NIHSS, ASPECTS, occlusion location, and time from onset to imaging. Second, IVT had a significant effect on EV (*p* = 0.01) after regression adjustment, with an adjusted EV of 6.9 mL (95% CI: 4.9–9.0 mL) for patients with direct EVT and 14.2 mL (95% CI: 9.0–19.5 mL) for patients with bridging EVT, adjusted for the aforementioned variables. In multivariable logistic regression analysis, IVT (OR: 2.78, *p* = 0.046), time from onset to imaging (OR: 1.34, *p* = 0.046), and ASPECTS (OR: 0.74, *p* = 0.044) were significantly and independently associated with sICH (Table 2, Figure 3). In multivariable linear regression analysis, IVT was significantly associated with higher EV (ß = 8.7, 95% CI: 2.6–14.8, *p* < 0.01) (Figure 4).

## 4. Discussion

The aim of this study was to investigate the impact of IVT on clinical outcome and secondary injury volumes in patients with complete endovascular recanalization compared to direct EVT. The main findings of this study are: (1) IVT was not associated with better functional outcomes compared to direct EVT but was (2) significantly and independently associated with increased rates of sICH and EV as secondary injury volumes. (3) Important predictors of outcome in TICI 3 recanalized patients were age and NIHSS, while ASPECTS, time from onset to imaging, or time from imaging to recanalization were not independent predictors, which emphasizes the importance of complete recanalization as major determinant of outcome.

In the light of the currently ongoing debate on whether to treat ischemic stroke patients with bridging IVT or direct EVT, the results of our study indicate that IVT may not be associated with better outcome, when EVT results in complete reperfusion. In the past, IVT has been associated with reduced lesion volume, as well as increased risk of secondary hemorrhage as a marker of blood brain barrier breakdown by destructive effects on the extracellular matrix and endothelial basal lamina [1,22]. Furthermore, IVT may be associated with neurotoxic cell injury in the ischemic brain by activation of excitatory aminoacid receptors [22]. In line with this, we observed a significantly increased EV after EVT in patients who received IVT, alongside the markedly increased rates of sICH, suggesting that IVT might be associated with increasing blood-brain barrier disturbance in this subgroup of patients. Previously, it has been observed that reperfusion in anterior circulation LVO patients may be associated with increased edema formation [23,24]. On the other hand, several studies showed that reperfusion is associated with reduced edema formation even in patients with low ASPECTS [25,26]. These ambiguous findings hint toward different courses of edema formation after thrombolytic and endovascular therapy and emphasize two important problems: a lack of understanding of the relationship between treatment and lesion pathophysiology, and the resulting consequences for optimization of individual treatment strategies.

Since approval of IVT for the treatment of ischemic stroke by the FDA in 1996, there has been an ongoing debate about harm and benefit of IVT administration and about the optimal treatment selection [27,28,29,30,31,32,33]. In 2018, the WAKE-UP trial reported a beneficial treatment effect of IVT compared to placebo despite a slightly increased rate of sICH [34]. This observation, however, lacked statistical significance in patients with an NIHSS > 10, which might be of importance considering the higher median NIHSS in the typical EVT eligible patient cohort (i.e., median NIHSS of 17 in the HERMES metaanalysis [2]).

More recently, the results of the DIRECT-MT, DEVT, and SKIP trial demonstrated non-inferiority of direct EVT compared to EVT + IVT, with a 4% CI margin in meta-analysis [35]. More importantly, all trials did not demonstrate that IVT increases the rate of successful EVT compared to direct EVT, which has been used as an important argument in favor of IVT administration [36,37,38]. In the DEVT trial, the proportion of patients with successful EVT (TICI 2b-3) and the end of the procedure was 88.5% for patients with direct EVT, and 87.2% for patients with bridging IVT, respectively, also showing no significant differences in times from onset to randomization, and time from onset to puncture. Second, the rate of reperfusion at follow-up was not different: 97% in patients with direct EVT, and 94% for patients with bridging IVT [37]. A further argument in favor of IVT was a supposedly improved microcirculation, and less peripheral emboli in new territories [39]. A recent study, however, observed that IVT had no effect on the number and volume of peripheral emboli after EVT independent of the degree of recanalization using high-resolution diffusion-weighted imaging [40].

Yet, the published results of the aforementioned trials on direct EVT versus bridging IVT still leave room for interpretation, and may not be sufficient to change clinical practice. Ethnical aspects might also contribute to differing results of the recently published studies, with an odds ratio (>1 favoring direct EVT) for the Asian trials (DIRECT-MT, DEVT, SKIP) of 1.08 (95% CI: 0.85–1.38) compared to 0.82 (95%: 0.63–1.07) in non-Asian trials (MR CLEAN noIV, SWIFT DIRECT). These differences could be suggestive of a different stroke etiology (i.e., atherothrombotic versus cardioembolic), which might yield a potential relevance for treatment selection. Nevertheless, the potential implications of an EVT only practice should be considered and discussed. Decision-making and workflow times could be significantly reduced, also considering the decline in primary stroke center admissions prior to transfer to an EVT center (“drip-and-ship”). Furthermore, the application of IVT is associated with high economical impact, with an approximate base payment of >12,000$ in the US [41]. Additionally, the administration of neuroproctants in combination with reperfusion might lead to further improvements in functional outcomes and could be tested as an alternative to bridging IVT [42]. In the ESCAPE-NA1 trial, the application of nerinetide was associated with better outcomes in patients who did not receive IVT [8]. Likewise, further drugs, such as glyburide, are currently tested as adjuvant treatment options [43,44].

To our knowledge, this is the first study that investigated the effect of bridging IVT on functional outcome and secondary injury volumes using quantitative imaging biomarkers in a patient collective exclusively consisting of complete reperfusion cases. Considering the rising frequency of successful vessel recanalization over time (e.g., approximately 60% in MR CLEAN, 72% in ESCAPE, 77% in DEFUSE-3, and 86% in ESCAPE-NA1 [2,8]), the importance of optimizing treatment strategies for these patients is suggested by the high proportion of patients with poor outcomes despite successful EVT as described in a recent meta-analysis (i.e., 45% mRS 3–6 in TICI 2b-3) [45,46]. The early identification of stroke patients who might not benefit from IVT could therefore be important to further improve functional outcome. The present study might give further insights into the effect of IVT on lesion pathophysiology in the setting of complete vessel recanalization, and might help to tailor individual adjuvant treatment options.

Limitations include the retrospective single-center nature of this study and the lack of a control group containing patients with incomplete reperfusion. This includes a potential selection bias regarding the indication of IVT which is caused by the study design. Finally, quantitative edema analysis by densitometry requires the absence of a space-occupying hemorrhage, is subject of time-consuming post-processing, and cannot be applied in patients with significant artifacts. A further limitation is the missing status of the cerebral collateral circulation, which might have an impact on secondary injury volumes. Furthermore, details on blood-pressure management, which might also affect the degree of secondary injury volumes, are unknown for this study. Future studies should analyze the identification of baseline variables to guide IVT, particularly in cases with high probability of vessel recanalization (e.g., time window, vessel anatomy, thrombus properties). Moreover, future research is necessary to investigate the impact of IVT in relationship to the degree of reperfusion for patients with posterior circulation stroke.

## 5. Conclusions

Bridging IVT was not associated with better functional outcome in patients receiving complete recanalization. The significantly increased edema volume and risk for sICH as secondary injury volumes could be a major reason for the lack of a clinical benefit of IVT in this patient cohort. The results of the present study support direct EVT approaches, particularly in patients with a higher likelihood of successful EVT.

## Figures and Tables

**Table 1:** Patient characteristics.

Baseline Characteristics	Bridgin IVT	Direct MT	*p* Value
Subjects, *n* (%)	128 (58)	91 (42)	
Baseline variables			
Age in years, median (IQR)	73 (62–80)	76 (66–83)	0.06
Female sex, *n* (%)	56 (44)	72 (56)	0.10
Admission NIHSS, median (IQR)	16 (12–20)	16 (10–20)	0.66
ASPECTS, median (IQR)	6 (6–8)	7 (5–8)	0.63
Time from onset to imaging in h, median (IQR)	3.1 (1.4–4.4)	2.0 (1.0–4–0)	0.38
Time imaging to reperfusion in h, median (IQR)	1.7 (1.3–2.0)	1.8 (1.6–2.8)	0.05
Endpoints			
Follow-up infarct volume in mL, median (IQR)	48 (18–92)	37 (11–65)	0.04
Follow-up NWU, mean % (SD)	14.7 (8.1)	11.9 (0.8)	0.02
Edema volume in mL, median (IQR)	6 (2–17)	4 (1–11)	0.04
ΔNWU, median % (IQR)	6.1 (0.5–11.9)	4.1 (1.6–7.7)	0.09
NIHSS at 24 h	10 (4–21)	13 (5–18)	0.75
Modified Rankin Scale, median (IQR)	4 (2–5)	3 (1–5)	0.61
mRS 0–2, *n* (%)	36 (28)	31 (34)	0.35
sICH, *n* (%)	26 (20.3)	7 (7.7)	0.01

NIHSS: National Institute of Health Stroke Scale, IQR: Interquartile Range, ASPECTS: Alberta Stroke Program Early CT Score, h: hours, mL: milliliters, NWU: net water uptake, sICH: symptomatic intracranial hemorrhage.

**Table 2:** Binary logistic regression analysis for good clinical outcome (A; mRS 0–2) and for symptomatic intracranial hemorrhage (B; sICH).

(A) mRS 0–2	Univariable Analysis			Multivariable Analysis			
	OR	95% CI	*p* Value	OR	95% CI		*p* Value
Age	0.94	0.92–0.97	<0.001	0.93	0.89–0.97		<0.001
NIHSS	0.82	0.77–0.88	<0.001	0.85	0.78–0.91		<0.001
Time onset to imaging	1.04	0.81–1.33	0.77	--	--	--	--
ASPECTS	1.20	0.98–1.47	0.004	--	--	--	0.26
IVT	0.76	0.42–1.35	0.35	0.48	0.21–1.12		0.09
Time image to recan	0.88	0.64–1.20	0.41	--	--	--	--
**(B) sICH**							
Age	1.00	0.98–1.03	0.65	--	--		--
NIHSS	1.03	0.98–1.09	0.25	--	--		--
Time onset to imaging	1.38	1.00–1.90	0.046	1.34	0.97–1.84	--	0.07
ASPECTS	0.74	0.55–0.99	0.045	0.73	0.54–0.99	--	0.044
IVT	3.06	1.27–7.39	0.01	2.78	1.02–7.56		0.046
Time imaging to recan	0.85	0.48–1.49	0.57	--	--	--	--

IVT: Intravenous treatment with alteplase.

## Data Availability

The data that support the findings of this study are available from the corresponding author upon reasonable request.
